# Association between preterm and low-birth weight with periodontal disease: a case-control study

**Published:** 2012-11

**Authors:** Nayyereh Khadem, Mohammad Ebrahim Rahmani, Alireza Sanaei, Mliheh Afiat

**Affiliations:** 1 *Women's Health Research Center, Department of Obstetrics and Gynecology, Imam Reza Hospital, Mashhad University of Medical Sciences, Mashhad, Iran. *; 2 *Department of Periodontology, Mashhad University of Medical Sciences, Mashhad, Iran. *; 3 *Dental Research Center, Mashhad University of Medical Sciences, Mashhad, Iran. *

**Keywords:** *Gum disease*, *Preterm delivery*, *Low birth weight*, *Gingivitis*, *Periodontitis*

## Abstract

**Background: **Since preterm delivery is an important problem in obstetrics, it is necessary to know the risk factors. Periodontal disease is an infectious disease and infection is risk factor for preterm delivery. Respecting to the mechanisms of preterm delivery and because involved mediators in this procedure are synthesized in periodontal disease, gum disease is investigated as risk factor for preterm delivery.

**Objective:** The aim of this study was to determine the association between preterm, low birth weight with periodontal diseases to improve the mothers’ and the children’s health.

**Materials and Methods:** This cases-control study was done on 70 women (mean age 25.01 yrs.) 35 women with preterm delivery, gestational age <37 weeks and birth weight <2500 gr as case group and 35 women with term delivery, gestational age >37 weeks and birth weight >2500 gr as control group referring to Imam Reza Hospital. Mean Probing Depth (MPD), percent of sites with more than 3 mm in probing, bleeding Index (BI), Plaque Index (PI), and Extent and Severity Index (Ext. and Sev.) were measured using a mirror and a standard William's periodontal probe.

**Results:** Significant difference was found in Mean Probing Deep (MPD), percentage of sites with more than 3 mm in probing, BI, PI, Ext. and Sev. indices in case and control groups. There was no significant difference in patient’s job, age, education, and husband’s job and education. No difference was observed between two groups in monthly income and gravidity.

**Conclusion:** Gum disease can be a risk factor for preterm delivery.

## Introduction

Prematurity and immaturity is a main cause of prenatal and infant mortality and morbidity in developed societies. In the United States, prematurity and the related disorders cause more than 70% of prenatal and infant mortality. Roughly, 28,000 infants lost their lives before the age of 1 in the United States in 2001, mostly (two third) due to preterm delivery (1). The proportion of children hospitalized at least once during the first year of life is almost 20%; and, the frequency of hospitalization is higher among children who were born weighing less than 2000 gr (2, 3). 

Prematurity leads to problems such as growth disorder, vision and hearing problems, cerebral palsy, respiration distress, sepsis, apnea, and retinopathy (4). Although no cause has yet been found for 25-50% of preterm deliveries, increasing evidences show that various infections play a part in undesirable fertility; infection, for instance, can be as a risk factor for preterm delivery of a low-birth-weight infant (5). Some findings have elucidated a high association between periodontal diseases (as an infective) and preterm delivery which was first indicated by Galloway *et al* in 1931 (6). 

Then Offenbacher (1996), Radnai (2002), Jarjoura (2005) and Dannon (2008) also showed the association between periodontal disease and preterm delivery (7-10). Another study indicated the cooperation of alternative prostaglandins and cytokines. Carta (2003) by measuring PGE2 and IL1β in gum groove found that the increase of such cytokines increases the preterm delivery rate and preterm rupture of the amniotic membrane (11). Moreover, researchers such as Tarannom and Crowther have proved that treatment of periodontal disease reduces the rate of preterm delivery and low birth weight (12, 13). Oral microbes are potentially infective to amniotic fluid and cause extra harm to some cases. 

Microbiological studies have demonstrated that infected amniotic fluid induces the inflammatory host immune response which activates proteins, interleukin-1 and tumor necrosis factor (TNF) that pass the embryo and consequently cause preterm delivery and low birth weight (14). Despite advances in obstetric care, preterm birth continues to be the leading cause of perinatal morbidity and mortality (15). 

This suggestion has led many investigators to seek evidence in this field (16-18). In general, preterm delivery and low birth weight and the related disorders have negative emotional and financial effects on the families and the society. Furthermore, the cost of treatment is high and the number of Neonatal Intensive-Care Units in Iran particularly in Mashhad is limited. 

Accordingly, this study was aimed to determine the association between preterm, low birth weight with periodontal diseases to improve the mothers’ and the children’s health.

## Materials and methods

This case-control study was conducted on the women referred to the Imam Reza Hospital from 2007-2008. Women with gestational age <37 weeks and birth weight< 2500g were considered as case group and women with gestational age >37 weeks and birth weight >2500 g as control group. 

In addition, the subjects suffering from any of following problems were excluded from the study: known bleeding diseases like idiopathic thrombocytopenic purpura, problems as a result of pregnancy in which there is bleeding and clotting problems like placental abruption and preeclampsia (systolic blood pressure >140 and diastolic >90), infection via the pulp lesion or having internal infection in any part of the body, systemic diseases like diabetes and urogenital system infections, history of abortion, stillbirth, and history of preterm delivery. The method of sampling was simple sampling.

Among the women referred to the obstetric ward of Imam Reza Hospital, 70 women were included in the study and divided into two groups of case and control. Post-delivery examination was performed 24-48 hours after delivery when the mother was recovered and periodontal examination possible. 

The patients filled out an informed consent form, which had previously approved by the research ethics committee of Mashhad University of Medical Sciences. They were informed that no risk threatens them in this study and also the examination process was explained to them. The research assistance, a gynecology resident, completed the form included demographic questions and medical history involving the patients’ age and education, husband’s job, is the husband a relative or not, monthly income, pregnancy age, the number of deliveries, amniotic sac rupture, bleeding when brushing the teeth, urinary and reproductive infection, and vaginal bleeding. 

Periodontal pocket depth: standard Williams’s periodontal probe was applied to measure the pocket depth using the Blind method in 6 sites of every tooth. The sites were as mesiofacial, midfacial, distofacial, mesiolingual, midlingual, and distolingual. Probe was gently inserted into the pockets; then the depth was measured and recorded. The researchers fulfilled the examination and the assistant recorded the information in the examination form. 

O’Leary Microbial plaque index (PLI): this is a simple technique for recording the superficial plaques (mesial, distal, lingual, buccal). At the examination time, a dental disclosing tablet was given to the patients. Having patients chewed the tablet and washed off the mouth, dyed surfaces were examined using the probe tip to find the soft accumulations in the dentogingival junction. Gingival bleeding index (GBI): this study applied Ainamo and Bay Index (1975) based on which the probe sulcus twisted. Any bleeding after 10 seconds was considered as the bleeding on probing and recorded in the examination chart. 

**Extent and severity index (ESI)**

a) Extent score is the number of examined sites in which the amount of attachment loss is more than 1 mm in the mouth. b) Severity score is the amount of attachment loss between the sites. Probing depth (PD): the amount of sites with more than 3 mm depth was measured in percentage and the association between the sites was assessed. 

**Statistical analysis**

Statistical analysis was performed using Man-Whitney, Tukey exact test and T-student test analyzed by SPSS software version 11.5; the p<0.05 was considered for significant differences.

## Results

A total of 70 pregnant women entered to the study; 35 women with gestational age <37 weeks and birth weight <2500 g were considered as case group and 35 women with gestational age >37 weeks and birth weight >2500 g as control group. In this study, pregnancy frequency, education, job status and income of the subjects and their spouses were analyzed in both case and control groups, considered with no significant difference. 

Then periodontal parameters including ESI, GBI, PLI and also the mean Probing depth (MPD) and percentage of the sites with probing depth of more than 3 mm for the full-mouth were evaluated. Mean and standard deviation of extent, severity, and bleeding indexes during probing; mean of probing depth, percentage of sites with probing depth of more than 3 mm for the full-mouth in both case and control groups are summarized in table I. 

There was a significant difference between case and control group in all above items. There is significant difference between two groups (p=0.007). Comparing the severity and bleeding indexes during probing regarding infant birth weight in both case and control groups are shown in table II. Based on data and the result of Mann-Whitney test, there was significant difference between severity and bleeding in low and normal birth weight infants. Based on data and the result of T-student test, in table III, there was a significant difference between extent indexes in low and normal birth weight (p=0.01); however, no difference was found between dental plaque indices in low and normal birth weight (p=0.01). 

**Table I:** Mean and standard deviation and average of extent, severity, and bleeding indexes during probing

	**Case group**				**Control group**			**p-value**
	**Mean**	**Std**	**Median**		**Mean**	**Std**	**Median**	
Extent index	21.5	15.5	18.3		6.8	8.95	2.50	0.0001*
Severity index	1.1	0.21	1.0		0.73	0.59	1.0	0.0001*
Bleeding index	13.4	9.9	10.7		6.05	8.9	3.4	0.0001*
Mean probing depth	2.99	1.21	2.78		2.11	0.42	2.09	0.0001*
Probing depth >3 mm (%)	10.75	10.19	9.1		2.6	4.4	0	0.0001*

* Mann-Whitney test.

**Table II:** Severity and bleeding indices during probing regarding infant birth weight

	**Birth weight**		**p-value**
	**Mean (<2500g)**	**Mean (≥2500g)**	
Severity index (SI)	45.41	28.49	0.001 *
Bleeding index (BI)	42.52	30.54	0.001 *

* Mann-Whitney test.

**Table III:** Dental plaque and periodontal disease indices regarding infant birth weight

	**Birth weight**		**p-value**
	**Mean** ** (<2500g)**	**Mean** ** (≥2500g)**	
Plaque index (PI)	7.26	7.2	0.007 *
Extent index (EI)	10.93	10.93	0.001*

* T-student.

## Discussion

In this study, periodontal parameters including GBI, PLI, PD, extent and severity index (ESI) found to be more considerable in case group than control group. Since these parameters indicate the gum health, it is concluded that control group had healthier gums than case group. Periodontal disease may cause preterm delivery because periodontal disease is infectious in origin and infection is considered as a risk factor for preterm delivery. Offenbacher *et al* (1996) showed that pregnant mothers with Clinical Attachment Loss (CAL) =3 mm and Periodontal Pocket Depth (POB) =60% are 7.5 times more probable to have low-birth-weight (≤2500g) new born (7). Furthermore, periodontitis in mothers is a risk factor for preterm delivery, as concluded in this study (7). 

Radnai *et al* (2006) also evaluated 41 women in case group with preterm delivery and 45 women in control group with normal delivery (8). In their study, BI and PD considered to be as ≥50% and >4mm, respectively; accordingly, the average weight of infants in case group was less than control group (p=0.047) (8). In another study, Jarjoura *et al* (2005) came across an association between the CAL and preterm delivery which was similar to our finding, considering that they evaluated the CAL of more than 3 mm and we evaluated the CAL of more than 1 mm (9). Furthermore, subjects with the probing depth of more than 5 mm were considered as patients in a study by Durtbudak *et al* (2005) which was technically similar to our study (14). 

It was found that there is a significant association between the percentage of periodontitis in both case and control groups and preterm delivery (p=0.001). In the above studies, parameters for periodontal disease were listed as PD average, AL, ESI and also in some studies PLI. Similarly, in the current study ESI, PLI, GBI, and percentage of sites with more than 3 mm (probing depth) were measured. In some studies, ESI was evaluated using Ramfjord teeth or it was evaluated in the one quadrant. For instance, Goepfert measured the ESI in one quadrant of women with average age of 23.9 yrs and found its significant association with preterm delivery (p=0.02) (19). However, this study conducted in full-mouth and with the evaluation of all parameters which resulted in more precise finding. 

The results of most similar researches were similar to this study. Nevertheless, Moore *et al* (1998-2001) found no association between periodontal disease and preterm delivery (20). Moreover, Lunardelli and Peres (2005) examined 449 women for the association between the mother’s periodontal disease and preterm birth with low birth weight, consistent with no statistical difference; periodontal pocket in four or more sites was considered as the disease indicator in their examination (21).

## Conclusion

Since case and control groups were statistically matched and there was a significant difference between two groups in gum health indicators, it can be concluded that gum disease may cause preterm delivery. Regarding the effects of periodontal disease on preterm delivery and low birth weight which cause prenatal and infant mortality, high treatment expenses, and the consequent emotional problems, we recommend the women to perform prophylactic activities, cure periodontal disease and care for tooth and oral health before and during the pregnancy. It also can be performed as a nationwide plan.
